# Charitable Infirmary

**Published:** 1831

**Authors:** 


### XXIV.

### CHARITABLE INFIRMARY.

ON SPINAL IRRITATION. By Dr. CORRIGAN.

WE are not sorry to find Dr. Corrigan in the field of practical medicine, instead of the labyrinth of the theory, or the deceptive quagmires of experiment. Although we have taken the liberty to differ from Dr. C. respecting the action and sounds of the heart, we shall be happy to meet him on less debateable ground—where cultivation will be sure to produce a good harvest.

In a clinical lecture delivered at the Charitable Infirmary, in Dublin, and published in our weekly contemporary, the LANCET, we find some interesting cases, with observations, of SPINAL IRRITATION, which we here propose to notice.

Dr. C. prefaces his observations on spinal irritation by remarking that morbid anatomy cannot be expected to throw much light on our investigations here, since the disease, though distressing, is seldom fatal—and since diseases of the nervous system do not always leave any visible trace of their existence, when death ensues.

"It fortunately, however, happens, that we have other means than those afforded by morbid anatomy, for ascertaining the seat of the disease, and by which we can, even during life, trace most distinctly the connecting link between cause and effect, or, in other words, between the diseased action and its symptoms. If we meet a patient complaining of hemicrania, or of pains in the neck and face resembling tic douloureux, and that we can produce or aggravate these distressing symptoms by pressure on the cervical vertebræ;—if we find another complaining of fits, of suffocative breathing, palpitation of the heart, and distressing sense of weight behind the sternum, and that we bring on, or aggravate, these symptoms by pressing on the dorsal vertebræ:—if we find another complaining of pains in the epigastrium and both hypochondria and globus hystericus, and of flatulent distention of the stomach, and that we can at any time bring on or aggravate all these by pressure on the lowest dorsal vertebræ;—if we find pains complained of in different parts of the body, in the right or left mamma, right or left hypochondrium, or in varied parts of the abdomen, and that pressure on the part of the spine generally corresponding in situation to the seat of the pain, aggravates the pain, or produces it if previously absent;—if we find, moreover, that local applications over the spine remove the distant symptoms or pains, not relieved by any other mode of treatment;—the fair inference from all this surely is, that the seat of disease is within the spine, and the connexion between the symptoms and the disease, between cause and effect, is, in such cases, more clearly exposed before us, than when even after death from acute disease, the knife of the pathologist exhibits to us some important lesion of structure, of which, rigidly speaking, the most we could say is, that it was probably the cause of the symptoms during life."

*Case* 1. This was a man, aged 27 years, a coach-painter, who was admitted on the 27th February into the Charitable Infirmary, complaining of flatulence, pain and tightness in the head and at the scrobiculus cordis, pallor and anxiety of countenance. The tongue was clean, pulse 90, respiration a little increased.

"There is pain along the course of the supra-orbital nerve of the left side. His sight is weak, and he cannot raise his eyes to any object above him, without experiencing pain and giddiness of the head. He is frequently attacked by 'globus hystericus.' He describes it as 'a ball of some kind' arising in his stomach, and ascending to his throat, where he feels it choking him; he also frequently experiences a sensation of tightness across his chest, which often prevents him from speaking when he wishes to do so. For the last two days he has had pain along the calf of the left leg, and weakness across his loins. His appetite is not impaired, but strength is much diminished. He has frequent pains across the epigastrium and both hypochondria, his nights are restless, and he starts often from his sleep, aroused by terrifying dreams; there is slight cough; epigastrium and both hypochondria are very tender under pressure, and pressure on the epigastrium produces instant cough and difficulty of respiration. The result of pressure along the spinal column is as follows; —pressure on any of the cervical vertebræ produces pain and lightness of the head; on the superior dorsal, produces tightness in the chest; on the inferior dorsal and first lumbar, produces pain at the scrobiculus cordis, flushing of the face, perspiration, and violent eructation of wind from the stomach; lower down produces no effect."

The patient was treated in a very simple manner. Rest, aloetic or oily purgatives; cupping, leeching, and blistering the spine, with the application of antimonial ointment, constituted the whole of the treatment, and the man was discharged cured on the 4th of April, or about 50 days from the time of his reception.

Without casting the shadow of a doubt on the facts of the above case, we cannot help expressing a little scepticism as to the inferred pathology of the disease. We have seen so many cases, especially in the female sex, where the above anomalous phenomena have presented themselves, but where the result disproved the cause to be spinal irritation, that we look on Dr. C.'s case with some distrust. The fact, however, is indisputable, that, in these cases, pressure on the spine often causes effects which are almost inconceivable. In the case of a young lady whom we have attended for some years, we were surprised to find that pressure on the two or three superior dorsal vertebræ produced convulsive motions of the lower extremities, and even of the abdominal muscles. We thought we had discovered a key to the host of nervous and hysterical symptoms with which this lady was harassed; but we were deceived. Horizontal posture, leeches, blisters, antimonial plasters, produced not the slightest relief, but rather aggravated the complaint. We afterwards found that a far less degree of pressure a little to the left of the epigastrium induced the same convulsive action of the muscles, as pressure on the spine—and even in a much higher degree. These phenomena are almost inscrutable. That they are referrible to the nervous system, we have no doubt; but that "SPINAL IRRITATION" conveys a true idea of their nature or seat, we are disposed to deny. And as to the *cause*, it will probably lead us astray. In the case to which we have just alluded, the presence of food, more especially any food of difficult digestion, in the stomach, produced not only twitchings of various muscles, but a strong disposition to *suicide*. So dreadful were the "MENTAL IMPRESSIONS," (the term applied by the patient,) that she nearly put an end to her own existence by starvation, to avoid the horrible feelings produced by food in the stomach! It is a curious fact, but one worth recording, that the medicine which was most operative in preventing these mental impressions, was five grains of the hydrargyrum cum creta taken an hour before dinner. The unhappy patient has often declared that, without the "grey powder," she had rather die than take food at dinner! We shall here introduce two or three other cases detailed by Dr. Corrigan.

*Case* 2. "The patient was a lady who, after her first confinement, suffered for several months from great weakness. When I saw her, about five months after her confinement, she complained of severe cough, difficult expectoration, and distressing sense of suffocation; these symptoms, particularly the last, much increased in the evening. There were constant pains in both hypochondria, in the epigastrium, and behind the lower third of the sternum. These pains were not increased by full inspiration, and only in scattered points of the right hypochondrium by pressure. Pressure on the last dorsal vertebræ aggravated them. Leeches were applied to the spine, and a recumbent posture was enjoined. On the next day she was much relieved of all her symptoms, and on the day following felt so very well, that she left the bed; and now the disease showed one of its most striking characters, viz. its connexion with change of posture. The exertion of dressing brought back all the symptoms in minor degree, although previous to rising from bed, no trace of the disease was observable. They again disappeared after short rest in a recumbent posture on a sofa. Rest was again enjoined, and the improvement again proceeded rapidly, convalescence being only interrupted by a walk a little too long, which, like the exertion of dressing already alluded to, produced a temporary aggravation of the symptoms."

*Case* 3. "The patient, a gentleman of about thirty-five years of years, complained of distressing paroxysms, which came on frequently in the course of each day, and often without any apparent immediate cause. The paroxysms commenced with a sensation of great distress, referred to some internal part behind the inferior third of the sternum, and rising from it upwards to the head; his eyes filled with tears, he became incapable of speaking, and his mental agony during the continuance of the fit was extreme. The paroxysm did not last more than a few minutes, and was then terminated by one or two heavy long-drawn sighs. The clapping of a door, an unexpected meeting with an acquaintance, or any similar trivial cause, was sufficient to bring it on. It seemed as if, in this case, the sympathetic ganglion, to which some refer the seat of the passions, was engaged, for pressure on the epigastrium was immediately followed by all the distressing symptoms already described. The third, fourth, and fifth dorsal vertebræ were tender, and pressure on them was attended by the same effects. A towel was dipped in warm water and passed slowly down the spine. The moment it came over the tender vertebræ, the same results followed as from pressure. The history of the case was this: About twelve months before, this gentleman suffered under much anxiety of mind from a weight of business; then, for the first time, he felt the sensations described, which never up to the time of my visit quitted him. The same treatment was prescribed as for the other cases."

*Case* 4. "A lady, æt. 50, came to town for advice with the following symptoms:—She felt severe pain about the umbilicus, and in the left lumbar region; this pain was always brought on, or greatly aggravated, by taking food; relief was obtained after a meal by lying down, and the pain which remained present during the day always ceased soon after retiring to bed at night; the abdomen was soft, and the digestive functions well performed; the spine had two lateral curvatures; there was a little tenderness, under pressure, of the two last lumbar vertebræ, but pressure on them did not bring on the pain of side. The history of the case was this: having previously suffered under great depression of spirits, consequent on the death of several of her children, this lady first felt, about three months before, some uneasy sensations about her shoulders, which obliged her to rest her back when knitting or sewing. These were followed by pain in the region of the liver, extending into the right hip, and, finally, all seemed to concentrate themselves in the intense pain of the left side already described. For the pain in the region of the liver she had been put under a course of mercury, and finally, from a fear that malignant disease of the stomach, or some of the organs in its immediate vicinity, was setting in, she was put on hemlock, and repeated blistering was ordered over the seat of the pain. All these measures failed to give relief. The first circumstance that made me look upon this case as one of spinal irritation, was the remarkable effect of posture. Moreover, there was not fever, nor wasting, in proportion to the intensity or the duration of the pain. Leeches were applied to the spine, and afterwards counter-irritation was kept up by tartar-emetic ointment. Two grains of sulphate of iron were at the same time ordered as a tonic, three times a day, and her bowels were kept open by Rufus's pill. Rest was of course enjoined for a few days. From that time up to the present, and several months have elapsed, there has not been a return of the pain in the slightest degree."

Our readers will form their own conclusions, as to the above cases, which are, however, very interesting, whatever theory may be employed for their explanation. For our own parts, we are much more disposed to attribute the phenomena to irritation of the ganglionic nerves, as distributed on the different viscera, than to spinal irritation. We advise Dr. Corrigan to prosecute the inquiry, and to attend to the state of the digestive organs, and of the uterine system, in females, when he will probably find cause to suspect the cause, if not the seat of the disease, in other parts besides the spine.

